# The implementation and refinement of a national institute for physical activity, health and sport

**DOI:** 10.1186/s12889-026-26524-z

**Published:** 2026-03-14

**Authors:** Amie B. Richards, Melitta A. McNarry, Catherine A. Sharp, James Shelley, Rachel L. Knight, Kelly A. Mackintosh

**Affiliations:** 1 Applied Sports, Technology, Exercise and Medicine (A-STEM) Research Centre, Swansea University, Swansea, Wales, UK; 2 Welsh Institute of Physical Activity, Health and Sport (WIPAHS), Wales, UK; 3 Policy and International Health, a World Health Organization Collaborating Centre on Investment for Health & Well-being, Public Health Wales, Cardiff, Wales, UK; 4 School of Health and Sport Sciences, Liverpool Hope University, Liverpool, England, UK; 5 Betsi Cadwaladr University Health Board, Ysbyty Gwynedd, Penrhosgarnedd, Bangor, Gwynedd LL57 2PW Wales, UK

**Keywords:** Co-development, Translational, Policy, Practice, Nationwide, Whole-systems

## Abstract

**Background:**

Recognition of the importance and value of collaboration and involving stakeholders throughout the research process has given rise to the establishment of the Welsh Institute for Physical Activity, Health and Sport (WIPAHS). Globally, formal collaborations between researchers and stakeholders to complete research projects, remains insufficient. Therefore, the primary aim of WIPAHS is to engage in long-term, forward-thinking collaborations that address practice-informed research questions, to disseminate findings widely, and translate them into practice and policy. This study explored the implementation and subsequent refinement of a national institute for physical activity, health, and sport.

**Methods:**

Through a qualitative exploratory design, thirteen, individual, semi-structured, online interviews were conducted with academic members of the WIPAHS Research Steering Group. The data were thematically analysed by the research team to identify themes and subsequently used to formulate a set of Principles of Working.

**Results:**

Fifteen sub-themes were generated which led to eight Principles of Working emphasising national unity and the commitment to working collaboratively across all of Wales, UK, for the benefit of Wales. The role of collaboration in amplifying real-world impact was particularly highlighted; collaborative efforts between academics and stakeholders, underpinned by a clear structure and equal opportunities, were believed to facilitate a reduction in research duplication and optimal efficiency.

**Conclusion:**

Formalising the developmental process and the resulting Principles of Working provides a replicable blueprint for other academics to consider creating similar national institutes. This approach enables co-ordinated, systems-wide efforts to promote and advance health-enhancing physical activity and sport, offering a model for others seeking a comprehensive approach to achieve these goals.

**Supplementary Information:**

The online version contains supplementary material available at 10.1186/s12889-026-26524-z.

## Background

Research partnerships yielding collaborations between Higher Education Institutions (HEIs) can build capacity for knowledge exchange and translation; [1] such endeavours offer valuable insights for addressing and resolving localised challenges [2]. Whilst the barriers hindering collaborations between HEIs and non-academic stakeholders (e.g. community, industry) have been explored, a stronger emphasis has been placed on developing inter-university teaching and learning collaborations than research. Stemming from the triple-helix model to research (university, industry, and government) [3], notable barriers for HEIs collaborating with non-academic stakeholders include: the concern that industry-related research may not align sufficiently with academic standards, such as ethics approval processes, posing challenges to publishing; [4] bureaucracy within HEIs (i.e. contract and legal negotiations); [5] the physical proximity and language barriers that researchers face [6], albeit now partially mitigated by technological advances; and, specifically within health research, concerns about impact and translation, or how to bridge-the-gap between research production and implementation [7]. 

In 2021, Universities Wales, a committee representing the interests of the eight HEIs in Wales, United Kingdom (UK), cited a requirement for Welsh Universities to create formal arrangements under which they could collaborate in research and innovation [8]. This requirement significantly aligned with the ethos of the Welsh Institute of Physical Activity, Health and Sport (WIPAHS), established in 2019, to: (i) enhance collaborative research capacity across Wales, beyond that of academia, including research with stakeholders; (ii) promote the health benefits associated with sport and physical activity; and (iii) facilitate the translation of research and evaluation findings to tangible individual and societal impacts; with the overall mission being to reduce inequalities in physical activity, health and sport, and increase physical activity levels, and subsequently health outcomes, in Wales. Currently (2024), the WIPAHS Research Steering Group (RSG) is comprised of University Representatives from all eight Welsh HEIs, and eight Strategic Theme Leads, made up of academics and practitioners, who are experts in their fields, ranging from policy to behaviour change, through to mental health and well-being; this multi-disciplinary composition of WIPAHS facilitates the transfer of knowledge and promotes multi-disciplinary work [9] (organisational structure available in Supplementary File 1.) The RSG works with stakeholders at all stages of research, to support and promote true collaboration, sharing authority and accountability throughout the entirety of the project. Relevant stakeholders should be actively engaged in HEI collaborations throughout the research process to help identify priorities, understand the problems being explored, and devise creative solutions [10]. With representatives from Sport Wales, Welsh Government and Public Health Wales on the Strategic Management Board (SMB), WIPAHS receives direction in line with the nations priorities and has a direct route into influencing practice and policy.

Whilst there is a plethora of examples of collaborations within research at an individual study level, such practices remain unexplored at a pan-institution level. To effectively learn how to develop partnerships and collaborations which yield positive outcomes at local, national, and global levels, and are primarily guided by non-economic motives [11], it is imperative that interdisciplinary collaborative experiences are drawn upon [12]. Knowledge and understanding surrounding collaboration ideology, including their design and implementation, is sparse [13, 14]. Despite the high value placed on national and non-academic sector collaborations [15], researchers rarely consider the formation of, and ongoing developments from, their collaborations [16]. Therefore, the aim of this study was to: (i) gather WIPAHS RSG members expert opinions on the formation and implementation of a national research collaboration; and (ii) to develop a set of guiding Principles of Working.

## Materials and methods

### Study design and participants

Using a qualitative exploratory design, semi-structured, online, individual interviews were conducted with members of the WIPAHS RSG (*n* = 13; 62% female). Participants were purposively recruited, via email, to take part based on their membership of the RSG (i.e. either as a HEI representative or as a Strategic Theme Lead). Therefore, the sample number reflects the number of people on the RSG at the time of the data collection (2022), excluding one Strategic Theme Lead and the chair of the RSG. Participant roles within WIPAHS or their employing HEIs are not reported alongside quotations to protect anonymity, as the small number of individuals in these positions could make them easily identifiable; further details of their roles are publicly available via the WIPAHS website and annual report. The interviews were conducted by a WIPAHS researcher (CAS) within the first three weeks of them starting their role at WIPAHS to minimise familiarity bias. The interviews were conducted and recorded using an online video conferencing software (Zoom Video Communications, San Jose, CA). One-hour slots were arranged with each participant to conduct the interview. Informed written consent and additional verbal consent were obtained prior to study commencement. The interview guide (Supplementary File 2) was designed by two researchers (CAS and JS) and refined following preliminary testing with two WIPAHS members. The guide was developed to explore the perceptions and experiences of participants in relation to their role and responsibilities within WIPAHS. Key areas of discussion included initial involvement with WIPAHS, thoughts on the structure and mission of WIPAHS, and overall perceptions. Institutional Research Ethics Committee approval was obtained from Swansea University (JS_01-09-21).

### Data analysis

Interviews were transcribed using the automated transcriber on Zoom© (Zoom Video Communications, San Jose, CA), in line with approval from IT services and institutional ethics, but were also manually checked, cleaned, and anonymised. The generated data were thematically analysed, drawing on the six-step approach outlined by Braun and Clarke [17, 18]. An inductive, data-driven, approach was used, facilitated by familiarisation, reading and re-reading the transcripts, and noting any data that was repetitive, novel, or related to previous research. NVivo 12© (QSR International, Melbourne, Australia) was used by the first author (ABR) to code the data and generate sub-themes. On completion of sub-theme coding, to enhance rigor, credibility, and transparency [19], a second author (RLK), also repeatedly read the transcripts, acted as a ‘critical friend’ to reflexively discuss, and challenge the generated sub-themes. This latter step was also important as all members of the study team had links to, and pre-existing knowledge of, WIPAHS, and therefore this step ensured that researcher bias did not influence generated sub-themes. Where required, codes and sub-themes were mapped back to the original data and refined in a recursive manner until an accurate and true representation was ensured. Following further collaborative discussion with the study team (KAM and MAM), two authors (ABR and RLK) mapped the generated sub-themes into higher-level themes, which have been termed ‘Principles of Working’.

## Findings

Fifteen sub-themes were generated, further refined, and synthesised into a set of eight Principles of Working of equal importance. Subsequent discussion of the findings are structured around these Principles and illustrated by direct quotes from the interviews (Table 1).

**Table 1:** Codes, sub-themes and subsequent higher-level themes termed principles of working

Principle of Working (Higher-Level Theme)	Sub Theme	Code
Smart Research, Solid Structure: Minimizing Duplication, Maximizing Efficiency.	Reduction of Duplication	Reduce duplication of research
	Structure	Theme lead role
		Researcher role
		HEI lead role
		ECR contribution
		Cross-discipline
Empower Research for Real-World Change: Make it Community-Driven, Make an Impact!	Real-World Impact	Translation of research
		Knowledge exchange
		Real world impact
		Dissemination
		Community-driven
	Policy	Policy
		Future Gen Act
Stakeholder Synergy: Partnering with a shared purpose.	External Partners	External partners
		Sport Wales/PHW/Welsh Gov
	Stakeholders	Stakeholder
Equal Footing, Empowered Voices: Championing Opportunity for All	Equal Voice	More vocal, everyone heard
		Equality (everyone heard) (for all)
	Equal Opportunities	Opportunities
		Open Involvement
		Project Allocation
		EOI’s
National Unity: Working across Wales, for Wales.	Nationwide	Country distribution
		Pan-Wales
		Welsh
	Welsh Focus	Relevance to Wales
		Research Questions
Unlocking Potential through Collaboration: Amplifying Our Impact.	Promotion of Collaboration	Collaboration
		Transparency
		Co-Production
		Different strengths/weaknesses (of different universities)
		Sharing
		Disconnect/conflict between organisations
	Greater Impact	Impact – bigger from collab working – publications/funding
		Benefit for researchers
		What’s in it for us?
Clear Mission, Infinite Horizons: Pioneering the Visionary Journey	Future Vision	Specific Objectives
		Future Vision
	Mission	Mission
An Iterative Cyclic Process of Dynamic Listening: Identifying Challenges and Taking Action	Potential Challenges	Time constraints
		Personal Agenda
		Recognition
		Funding
		WIPS Confusion
		Awareness of WIPAHS
		Perceived Swansea Based
		Size of WIPAHS
		Sustainability

### Principle 1: smart research, solid structure: minimising duplication, maximising efficiency

This principle amalgamated two themes: ‘Structure’ and ‘Reduction of Duplication’. Having a solid, well-defined, comprehensive structure, of both the institute itself and how it operates, which is understood by all members, was considered essential to enhance operational efficiency and minimise research duplication. Reducing duplication and not *‘reinventing the wheel*,* if something’s already done’* (Participant 8) was also a stand-alone discussion topic. Participants cited that a key strength was that WIPAHS aims to reduce research duplication through actively raising research activity awareness across the HEIs through scheduled, formalised discussions alluding to the *“removal of competing projects which are underpowered and can’t answer the research questions… so making our work more efficient”* (Participant 6). Moving from duplication of small, isolated studies, towards collaborative, larger-scale, more robust, and higher-powered studies, will not only enable additional expertise but account for inter-geographical area and demographical nuances.

### Principle 2: empower research for real-world change: make it community-driven, make an impact!

This principle reflects two themes: ‘Real-World Impact’ and ‘Policy’. Participants unequivocally articulated the need for WIPAHS to get “*academics to understand how the research will translate into the real-world”* (Participant 1) and how real-world impact should be *“the heart of what we do… almost all of the work that WIPAHS does has got to be based on the idea that it’s going to go into the real world… we always ultimately should be thinking*,* how will that translate into practice”* (Participant 1); with this being achieved by providing tangible, practical benefits for collaborators and stakeholders through to increased influence on policy. Indeed, “*given the vision*,* we need to be listening to the needs of our communities. Those are the real novel issues*” (Participant 7). This emphasis on listening and responding to community needs reinforces how WIPAHS’s influence on policy can be strengthened by prioritising issues that resonate within these communities, ensuring that research outputs not only inform policy but also reflect, and address, the lived experiences and priorities of those affected.

### Principle 3: stakeholder synergy: partnering with a shared purpose

Participants stressed the value of connecting directly with *“external stakeholders to make sure what we do is relevant to them”* (Participant 13), to understand their insight needs, draw on their experiences to inform the approach, and support translation into practice and thus impact. Participants highlighted the need to be *“engaging stakeholders to really ensure we’re looking at the right things and having an impact*” and “*asking the right questions in the right ways and having the right people involved in the research”* (Participant 2). Collaborations should endeavour to *“bridge the gap… a little bit more between these stakeholders and academia”* (Participant 5) and other sectors (e.g. private sector, public sector) to incorporate a wider range of partnerships; with people from multiple sectors collaborating on topics of interest. Indeed, WIPAHS has integrated members over time and has representatives from Welsh Government, Public Health Wales, and Sport Wales on the Strategic Management Board, giving researchers the opportunity to widely disseminate research and translate this into meaningful outcomes through these bodies.

### Principle 4: equal footing, empowered voices: championing opportunity for all

This principle is derived from the theme ‘Equal Opportunities’, reflecting aspects such as equality, opportunities, open involvement and project allocation. Deemed paramount within this principle was the importance of being *“super transparent… super open and fair”* (Participant 10) when communicating opportunities presented or identified by WIPAHS, and any decisions made. Whilst it was acknowledged that *“not every university is going to be able to contribute to every piece of work that is offered*,* but as long as there’s the opportunity that they can”* (Participant 13) regardless of HEI size or geographical location, “*enabling a voice for all”* (Participant 12), and thereby including non-academics, was fundamental.

### Principle 5: national unity: working across Wales, for Wales

This principle was created from two themes: ‘Nationwide’ and ‘Welsh Focus’. The discussion highlighted the uniqueness of a pan-Wales network operating at a national level where “*geographical spread… allows for a broader reach in terms of research and representation within research so that it reflects the entirety of Wales”* (Participant 14) was emphasised, along with how to effectively harness the power of collaboration across Wales, to benefit the population. A predominant feeling that “*collectively*,* theoretically*,* we should be stronger as a collaboration”* (Participant 5) ensued and that *“rather than work in sort of isolation and independently from one another… we can work so much better and have better results that demonstrate what Wales can do*,* what Wales can offer”* (Participant 13). It was also believed that consolidating localised knowledge and skills, drawing on the *“great amount of expertise*,* creativity in innovativeness across universities in Wales”* (Participant 13) improved the work undertaken, and that there was value in exploring how to *“publish stuff that is actually relevant to Wales and then try and see whether we can make wider parallels and recommendations for… the societies that are similar to Wales”* (Participant 11). Thereby, the work extends to having “*more international audiences… that could potentially have a straightaway much bigger impact for Wales”* (Participant 11), facilitating international comparability and collaboration.

### Principle 6: unlocking potential through collaboration: amplifying our impact

Participants felt that the ‘Promotion of collaboration’ between researchers, practitioners, policy-makers and the community was unique within WIPAHS and much needed in the scope of Higher Education and research more generally. Whilst *“WIPAHS is brilliant”* (Participant 7) in fostering early signs of a collaborative culture amongst the HEIs, with the initial indications of the advantages of collaboration evident, some participants voiced concerns and caution that *“people don’t run away and take advantage of”* (Participant 7) sharing and owning research ideas. Moreover, partnering with researchers and stakeholders across Wales resonated positively with many, identifying opportunities extending beyond networking, to maximise the impact of WIPAHS and, indeed, enabling individual researchers *“to engage in applied research and have a wider impact over and above what [they] could do on [their] own”* (Participant 7), generating more higher-quality outputs from collaborative endeavours. Overall, WIPAHS was perceived as *“pulling together… the body of researchers in Wales”* and being a *“vehicle to try and pull things together in a more strategic*,* coherent way”* (Participant 10), whether that was by *“putting in grants or working on publications*,* so that we can have an impact on public health policy”* (Participant 14). Ultimately, the transparent WIPAHS model of collaboration can facilitate higher quality research endeavours, and thus greater impact, on the health and well-being of Wales.

### Principle 7: clear mission: pioneering the visionary journey

The concept of the ‘mission’ of WIPAHS was discussed in the interviews, with in-depth conversations revolving around specific objectives and future visions. This dialogue was guided by interview questions regarding the direction and sustainability of WIPAHS. The consensus that WIPAHS’ “*purpose largely focused on physical activity”* (Participant 8) was expanded by views that it was also about *“making a difference and trying to influence collaborations across Wales”* (Participant 9). Whilst others highlighted the WIPAHS’ mission as being *“to generate the evidence base for what is actually happening with physical activity”* in Wales and the *“impact on people’s ability to thrive and flourish”* (Participant 7). Emphasis was placed on the requirement to transparently communicate, and, where necessary, reinforce the ‘mission’ to ensure that the remit of WIPAHS remained aligned, minimising any divergence towards individual endeavours, and those of the respective HEIs that may potentially confound WIPAHS’ overarching ‘mission,’ potentially due to employment being through the HEIs and involvement in WIPAHS being on a voluntary role basis, *“I’m conscious that I don’t want my personal agenda to influence the agenda of [WIPAHS]”* (Participant 14).

### Principle 8: an iterative process of dynamic listening: identifying challenges and taking action

This principle was created from discussions around acknowledging the ‘Potential Challenges’ that WIPAHS faces, both as a network and from the perspective of the individuals involved with WIPAHS; highlighting the need to engage in an iterative process of actively listening to, and addressing, concerns to ensure continued progress. Such challenges included time constraints and funding availability: *“I think it’s always hard when people volunteer for roles and there’s no payments… so you are relying on goodwill”* (Participant 12); awareness of WIPAHS and ensuring its long-term sustainability: *“I would like to see some targets and objectives for what we want from the research; what do we want to see from research and the next five years. I’d like to see it [WIPAHS] just really widely known*,* I’d like to see it just everyone knowing about it*,* and everyone linking to it”* (Participant 1).

## Discussion

This study sought to involve WIPAHS members in a formative process to inform the ongoing development of a national institute of physical activity, health, and sport. The results yielded a guiding set of eight essential Principles of Working, which are crucial for informing the continuous growth, and indeed the successful establishment of other similar national entities. Principles revolved around: transparency, openness, collaboration, maximising real-world impact, engaging stakeholders, minimising research duplication, and fostering national unity.

Considering the first Principle of Working, participants discussed WIPAHS’ role in encouraging the sharing of data, methodologies, and findings, to reduce duplication and promote greater efficiency within research. Duplication of research not only consumes valuable time and funding but also delays scientific progress, whereas collaboration enhances research productivity [20]. By fostering a culture of co-ordination and collaboration, researchers and institutions can consolidate their collective expertise and resources [21] to address critical research questions more effectively and allocate research funding more efficiently; [22] whilst reducing bureaucratic inefficiencies [23]. 

Naturally, some of the Principles of Working overlapped, such as Principles Two and Three, regarding having a real-world impact and the importance of stakeholders, which, according to Brownson et al. [24], is where the true value of research is realised (i.e. translating research into tangible benefits for society). Working with stakeholders is crucial as it increases the likelihood that research outcomes have real-world relevance, through integrated knowledge translation [25]; but is not without bureaucratic challenges [5]. The interviews highlighted stakeholders as key to the WIPAHS network, due to their ability to bring valuable insight and real-world questions that need solutions. Indeed, Kothariet al. [26] identified stakeholders as key to enhancing the quality and applicability of research findings, practical knowledge, and contextual expertise to the research process. This is exemplified by numerous WIPAHS projects, available in the WIPAHS Annual Reports, such as a recent collaboration with a health board to investigate healthcare professionals’ approaches to promoting physical activity in a clinical paediatric population (Supplementary File 3). The findings have been shared with the stakeholder, who is now co-ordinating discussions across Wales to streamline and standardise these practices nationwide. Engaging stakeholders with real-world questions fosters a reciprocal relationship, where researchers address community-identified priorities and challenges, and stakeholders gain confidence in academic findings to drive meaningful change.

Equality, diversity, and inclusion (EDI) are central to a functioning society [27], which are reflected in Principle Four. Equal access and participation in a network enables individuals from various backgrounds to contribute their unique perspectives and experiences, leading to the enrichment of ideas and solutions [28]. Furthermore, equitable networks strengthen social cohesion [29]. In essence, the provision of equal opportunities within networks should reflect a thriving research network. Central to the effectiveness of WIPAHS’ work is the assurance of adequate representation, ensuring that outcomes are not only translatable but also reflective of the entire population. Ultimately, the principles of EDI within WIPAHS are the foundation of our commitment for, and use of, collaboration within research, to hear the voices of the people in Wales. Integral to this, is providing a national approach within the network; pan-Wales working is of paramount importance for the collective well-being of the people of Wales (Principle Five). Such an approach allows for the efficient utilisation of resources and expertise (highlighted in Principle One), promoting the development of consistent and co-ordinated strategies that can benefit individuals and communities across the country [30]. A national network can help in addressing common challenges [31] and harnessing collective strengths, whilst encouraging inclusivity and promoting a sense of shared research identity [32]. Ultimately, the national approach encompassed by WIPAHS strives to create a more prosperous, equitable, and harmonious Wales for all its residents [33]. The Well-Being of Future Generations (Wales) Act [33] outlines five ways of working: Integration, Collaboration, Involvement, Prevention and Long-Term. The findings from this study indicate that WIPAHS actively aligns with these principles, demonstrating a commitment to fostering a positive and enduring impact on future generations.

This study demonstrated that collaboration is highly valued and plays a pivotal role in advancing knowledge across various disciplines and enabling greater impacts to be realised, as encompassed within Principle Six. A collaborative approach, if done well, facilitates the exchange of diverse perspectives, expertise, and ideas to enrich the research process [34] including greater levels of dissemination and subsequent impact, through co-authorship outputs [35]. However, a pan-national, multi-HEI collaboration does not go without its challenges, such as coordinating efforts across HEIs with differing priorities and resources, managing communication among diverse teams, and aligning goals and expectations [34]. However, the development and formalisation of collaborations, such as WIPAHS, provides a unique and powerful opportunity for individuals and HEIs to leverage their strengths leading to more comprehensive approaches to solving complex societal issues [36]. 

In accord with George et al. [37]. participants identified that, whilst they have a shared vision within WIPAHS, clear objectives are needed to ensure the mission of WIPAHS is not lost, as reflected in Principle Seven. Having a clear mission and well-defined objectives within a network are crucial for guiding its purpose, focus, and actions effectively [37]. Clarity in objectives provides a shared understanding of the network aims, enabling members to align their efforts and resources, and ensuring that everyone is working towards a common vision [37]. Indeed, WIPAHS has continued to evolve since the interviews, and now consults the Strategic Management Board to ensure that the WIPAHS vision and mission remains aligned to the key goals and activities of Public Health Wales, Welsh Government and Sport Wales.

This is the first study to explore the creation and refinement of a national institute of physical activity, health, and sport. The qualitative, online interviews allowed for a greater insight into the implementation and ongoing development of WIPAHS. However, the study is not without limitations. There was an inherent potential for social desirability bias as the researcher who conducted the interviews (CAS) was also part of the WIPAHS working group. Nonetheless, this was mitigated by several measures, including: (i) the interviews being conducted only three weeks into the researchers WIPAHS role; (ii) by having an a priori interview guide, ensuring anonymity and confidentiality in the reporting; (iii) the inclusion of a critical friend (a researcher who supported in data analysis; RLK); and (iv) the affording of equal importance to the themes and codes triangulated from each interview data set [38]. Whilst the study focussed on the academic collaborations within WIPAHS, thereby restricting the sample size, this has enabled a thorough understanding of the dynamics, strengths and challenges of such collaborations, which, in future research, can be verified and built upon with stakeholders.

## Conclusion

In conclusion, WIPAHS exemplifies an innovative model for addressing complex challenges in research collaboration and knowledge translation. By formalising a pan-Wales approach, WIPAHS unites diverse academic institutions and stakeholders to establish a clear structure that seeks to minimise research waste, fosters equal opportunities, and amplifies real-world impact. Its Principles of Working reflect a commitment to national unity and inclusive collaboration, ensuring that research questions are community-driven and needed in the ‘real-world.’ WIPAHS’ unique design, built on systems-wide collaboration, has transformed traditional research practices into a co-ordinated effort for societal betterment. The findings from this study offer a replicable framework for developing similar national institutes, providing actionable insights into overcoming challenges in collaboration and creating enduring impact across regions and disciplines.

## Supplementary Information

Supplementary Material 1.

Supplementary Material 2.

Supplementary Material 3.

## Data Availability

Full interview transcripts cannot be provided, due to identifiable data, despite being anonymised; descriptions of locations and activities mean that the participants could potentially be identified.

## Abbreviations

WIPAHS: The Welsh Institute of Physical Activity, Health and Sport
HEI: Higher Education Institution
UK: United Kingdom
RSG: Research Steering Group

## References

[1] Ayah R, Jessani N, Mafuta EM. Institutional capacity for health systems research in East and central African schools of public health: knowledge translation and effective communication. Health Res Policy Syst. 2014;12:20. 10.1186/1478-4505-12-20 PMC4064507 24890939

[2] EinterzRM, Kimaiyo S, Mengech HN, Khwa-Otsyula BO, Esamai F, Quigley F et al. Responding to the HIV Pandemic: The Power of an Academic Medical Partnership. Academic Medicine. 2007;82(8):812–8. 10.1097/ACM.0b013e3180cc29f1. 10.1097/ACM.0b013e3180cc29f1 17762264

[3] Leydesdorff L, Etzkowitz H. Conference report The Triple Helix as a model for innovation studies. Vol. 25, Science and Public Policy. 1998. Available from: https://academic.oup.com/spp/article/25/3/195/1630936

[4] Tartari V, Salter A, D’Este P. Crossing the rubicon: exploring the factors that shape academics’ perceptions of the barriers to working with industry. Camb J Econ. 2012;36(3):655–77.

[5] Salimi N, Bekkers R, Frenken K. Success factors in university-industry phd projects. Sci Public Policy. 2016;43(6):812–30.

[6] Hoekman J, Frenken K, Tijssen RJW. Research collaboration at a distance: changing Spatial patterns of scientific collaboration within Europe. Res Policy. 2010;39(5):662–73.

[7] Boaz A, Hanney S, Borst R, O’Shea A, Kok M. How to engage stakeholders in research: design principles to support improvement. Health Res Policy Syst. 2018;16(1):60. 10.1186/s12961-018-0337-6 PMC6042393 29996848

[8] Reid G. Strength in Diversity. 2021.

[9] Avdeev S. International collaboration in higher education research: A gravity model approach. Scientometrics. 2021;126(7):5569–88.

[10] O’Cathain A, Croot L, Duncan E, Rousseau N, Sworn K, Turner KM et al. Guidance on how to develop complex interventions to improve health and healthcare. BMJ Open. 2019;9e029954. 10.1136/bmjopen-2019-029954 PMC6701588 31420394

[11] Nguyen PM, Elliott JG, Terlouw C, Pilot A. Neocolonialism in education: cooperative learning in an Asian context. Comp Educ. 2009;45(1):109–30.

[12] Sewankambo N, Tumwine JK, Tomson G, Obua C, Bwanga F, Waiswa P et al. Enabling dynamic partnerships through joint degrees between Low- and High-Income countries for capacity development in global health research: experience from the Karolinska Institutet/Makerere university partnership. PLoS Med. 2015;12(2):e1001784 . 10.1371/journal.pmed.1001784 PMC4315568 25646629

[13] Wiek A, Bernstein MJ, Laubichler M, Caniglia G, Minteer B, Lang DJ. A global classroom for international sustainability education. Creat Educ. 2013;04(04):19–28.

[14] Caniglia G, John B, Bellina L, Lang DJ, Wiek A, Cohmer S, et al. The glocal curriculum: A model for transnational collaboration in higher education for sustainable development. J Clean Prod. 2018;171:368–76.

[15] Tetrevova L, Vlckova V. Collaboration between higher education institutions operating in the Czech Republic and the Non-Academic sphere. Eur Educ. 2020;52(1):68–79.

[16] Yokoyama K. Reflections on the field of higher education: time, space and sub-fields. Eur J Educ. 2016;51(4):550–63.

[17] Braun V, Clarke V. Using thematic analysis in psychology. Qual Res Psychol. 2006;3(2):77–101.

[18] Braun V, Clarke V. Reflecting on reflexive thematic analysis. Qualitative Research inSport, Exercise and Health. 2019;11(4):589–597. 10.1080/2159676X.2019.1628806.

[19] Smith B, McGannon KR. Developing rigor in qualitative research: problems and opportunities within sport and exercise psychology. Int Rev Sport Exerc Psychol. 2018;11(1):101–21.

[20] Abramo G, D’Angelo CA, Solazzi M. The relationship between scientists’ research performance and the degree of internationalization of their research. Scientometrics. 2011;86(3):629–43.

[21] Heinze T, Shapira P, Rogers JD, Senker JM. Organizational and institutional influences on creativity in scientific research. Res Policy. 2009;38(4):610–23.

[22] Kyvik S, Reymert I. Research collaboration in groups and networks: differences across academic fields. Scientometrics. 2017;113(2):951–67. 29081555 10.1007/s11192-017-2497-5 PMC5640763

[23] Leite D, Pinho I. Evaluating collaboration networks in higher education research. Cham: Springer International Publishing; 2017.

[24] Brownson R, Colditz G, Proctor E. Dissemination and implementation research in health: translating science to Practice. Second. New York: Oxford University Press; 2018.

[25] Kothari A, McCutcheon C, Graham ID. Defining integrated knowledge translation and moving forward: a response to recent commentaries. International Journal of Health Policy and Management. 2017;6(5):299–300. 10.15171/ijhpm.2017.15. 10.15171/ijhpm.2017.15 PMC5417154 28812820

[26] Reed MS, Stringer LC, Fazey I, Evely AC, Kruijsen JHJ. Five principles for the practice of knowledge exchange in environmental management. J Environ Manage. 2014;146:337–45. 25194520 10.1016/j.jenvman.2014.07.021

[27] Wolbring G, Nguyen A, Equity/Equality. Diversity and Inclusion, and other EDI phrases and EDI policy frameworks: A scoping review. Trends High Educ. 2023;2(1):168–237.

[28] UK Research and Innovation. UKRI's Equality, Diversity and Inclusion Strategy: Research and Innovation by Everyone, for Everyone. Strategy document. Swindon: UK Research and Innovation; 2023. https://www.ukri.org/publications/ukris-equality-diversity-and-inclusion-strategy/. Accessed 6 February 2026.

[29] Forret ML, Dougherty TW. Networking behaviors and career outcomes: differences for men and women? J Organ Behav. 2004;25(3):419–37.

[30] Kristoffersen ES, Bjorvatn B, Halvorsen PA, Nilsen S, Fossum GH, Fors EA, et al. The Norwegian praksisnett: a nationwide practice-based research network with a novel IT infrastructure. Scand J Prim Health Care. 2022;40(2):217–26. 35549798 10.1080/02813432.2022.2073966 PMC9397441

[31] Sonnenwald DH. Annual Review of Information, Science and Technology. In: Cronin B, editor. 2007. Available from: http://europa.eu.int/comm/

[32] Conner TW. Representation and collaboration: exploring the role ofshared identity in the collaborative process. Public Adm Rev. 2016;76(2):288–301.

[33] Welsh Government. Well-being of Future Generations (Wales) Act 2015. Legislation. Cardiff, UK: Welsh Government; 2015. https://www.legislation.gov.uk/anaw/2015/2/contents/enacted. Accessed 6 February 2026.

[34] Cummings JN, Kiesler S. Coordination costs and project outcomes in multi-university collaborations. Res Policy. 2007;36(10):1620–34.

[35] Katz JS, Martin BR. What is research collaboration? Res Policy. 1997;26:1–18.

[36] Cooke NJ, Hilton ML, editors. Enhancing the Effectiveness of Team Science. Washington. DC: The National Academies Press; 2015. 10.17226/19007. 26247083

[37] George B, Walker RM, Monster J. Does strategic planning improve organizational performance? A Meta-Analysis. Public Adm Rev. 2019;79(6):810–9.

[38] Nowell LS, Norris JM, White DE, Moules NJ. Thematic analysis: striving to Meet the trustworthiness criteria. Int J Qual Methods. 2017;16(1).

