# Supplementary figures and images for: The implementation and refinement of a national institute for physical activity, health and sport
Source: BMC Public Health. 2026 Mar 14;26:1313. doi: 10.1186/s12889-026-26524-z (PMC13101313; doi:10.1186/s12889-026-26524-z)

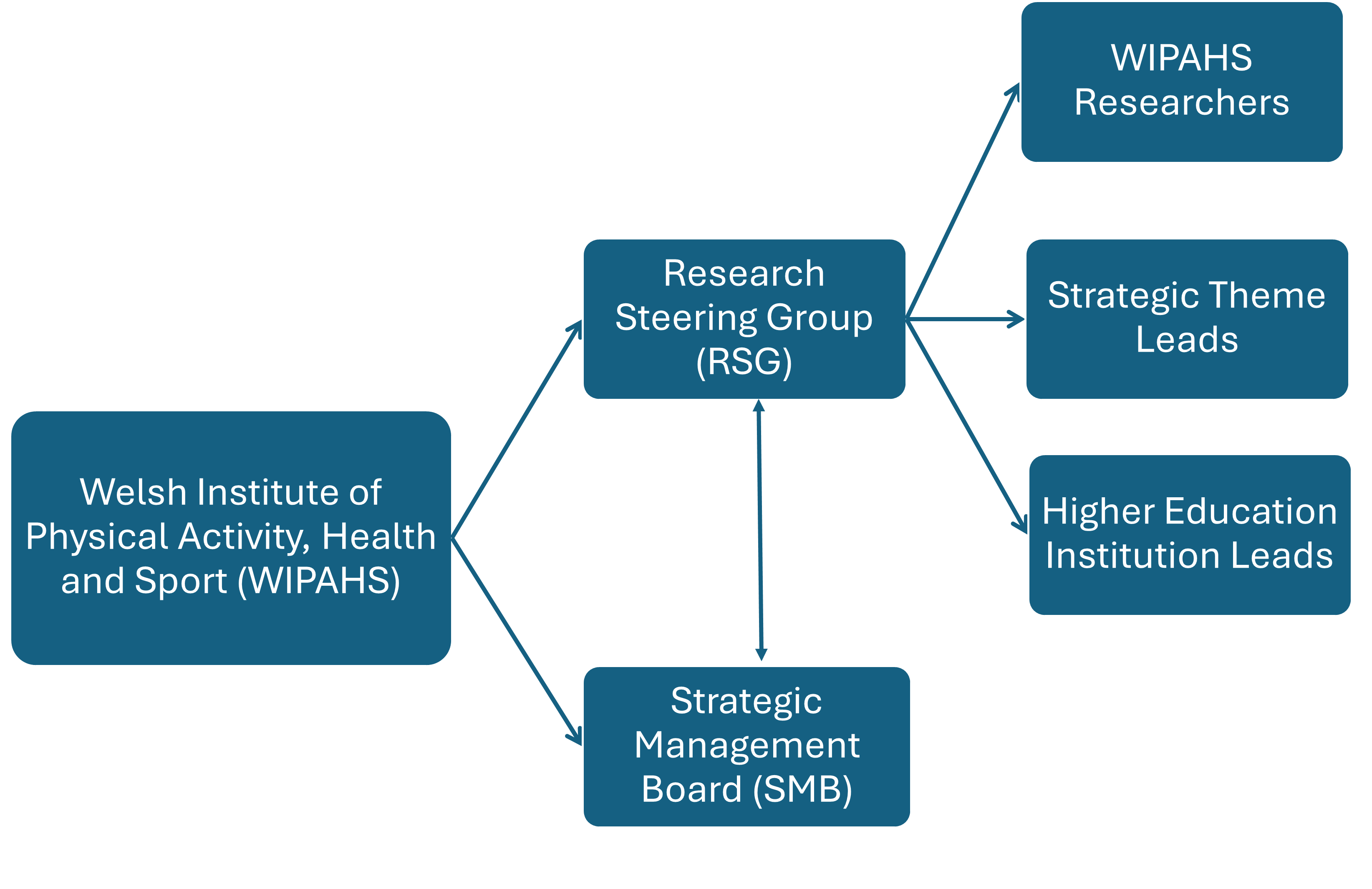

Supplement: Supplementary file 1 — Supplementary Material 1. [file 12889_2026_26524_MOESM1_ESM.png]
