# Supplementary material for: The implementation and refinement of a national institute for physical activity, health and sport
Source: BMC Public Health. 2026 Mar 14;26:1313. doi: 10.1186/s12889-026-26524-z (PMC13101313; doi:10.1186/s12889-026-26524-z)
Supplement: Supplementary file 2 — Supplementary Material 2. [file 12889_2026_26524_MOESM2_ESM.pdf]

SF1 - Example interview guide

The questions provided are examples of the topics to be discussed, although the exact questions may vary between interviews depending on the responses provided.

Introductory statement
Thank you for taking part in the interview today. As outlined in the materials shared beforehand, we are interested in your perspective on the establishment of WIPAHS and its next steps. The interview will broadly cover:<br>- Reflection on the progress of establishing WIPAHS; and<br>- How WIPAHS can best be integrated into established systems in Wales to meet its aims and mission.<br><br>There are no right or wrong answers. Please give as much information as possible, using examples where possible. I would also like to remind you that with your consent this interview will be recorded and transcribed for analysis. Any names or phrases that you use which could identify you will be coded or anonymised in the transcripts and reports so that you cannot be identified. Do you have any questions?<br><br>*Before we begin, please can I ask you to read the sentence on the screen to provide a verbal record of your verbal consent. This will be saved separately to your interview. [Press record]. Ready?*<br><br>*"I [NAME] consent to being involved in this research study and am aware of what is being asked of me and how my data will be used. I have been given the opportunity to ask any questions I would like to and am aware that I can withdraw from the study at any time without penalty."*<br><br>[Save consent recording]<br><br>Thank you, I will now begin the interview recording.<br><br>[Start interview recording]

Topic/Theme	Main Question	Sub-question(s)	Prompts
**[Transition statement]**<br><br>To provide context to your responses...			
Person	Please can you outline your current role in WIPAHS?		
	How long have you been involved in WIPAHS?		Years/Months?
	Briefly describe how and why you became involved in WIPAHS.		
**WIPAHS**			
**[Transition statement]**<br><br>*As you'll know, the mission of WIPAHS is is to facilitate the active involvement of key audiences in the identification of important research questions related to the health and well-being of the nation's future generations and to co-design research strategies to address them.*			

Purpose	When you joined WIPAHS, what did you feel was its purpose and mission?		Anything else to add?
	Do you feel that purpose and mission have been reflected in the work of WIPAHS?		
	Do you still feel WIPAHS has the same purpose or has your perspective changed?		Anything further?
Structure	What is your current understanding of how WIPAHS is organised and structured?		
	How do you think WIPAHS could be organised and structured to maximise its impact and reach?		Personnel (Zero-hour contract researchers?)<br>Structure<br>Succession planning?<br>ECR system?<br>Meeting setup (f2f / online)
	How does WIPAHS ensure balanced representation within the group, including on the Strategic Management Board?	What type of stakeholders do you think should be involved in decision making processes at the SMB?	
**[Transition statement]**<br><br>Great thank you, that's really interesting. A strength of WIPAHS is that it involves all 8 HEI's in Wales…			
HEIs	What do you think are the potential benefits for WIPAHS of having all HEIs involved?		
	What do you think are the potential weaknesses for WIPAHS of having all HEIs involved?		
	How do you think your institution can contribute to WIPAHS?		Infrastructure?<br>Collaboration?<br>Grant income?<br>Outputs?<br>Impact?<br>Geography?
	What are the potential benefits for your HEI to be involved in WIPAHS?		

	What are the potential weaknesses for your HEI to be involved in WIPAHS?		
	How can WIPAHS support your institutions involvement?	How do we break down the barriers for researchers to share their research?	Institutional defence barrier?<br>Equity in institutional kudos.
	How do we better share information and opportunities beyond institutional leads / strategic theme leads?		Any particular methods or techniques?
	What do you see the role of the institutional leads to be?		

[Transition statement]

Thank you for your insight. Next I would like to ask you about the strategic themes.

Strategic Themes	How well do you feel the strategic themes align with the purpose of WIPAHS?	How often do you think they should be reviewed?	
	How were the themes generated and do you think they are appropriate?	Should the same process or a different process be used when they are reviewed?	
	What do you see the role of strategic theme leads to be?		

[Transition statement]

Great thank you. Now to consider external stakeholders…

**Stakeholders**	What are the benefits for [your] organisation to work with WIPAHS?		

[Transition statement]

Fantastic, thank you. I would now like to ask you about how WIPAHS integrates into Wales more broadly.

**Process**	How do we develop research within the WIPAHS group and how do we allocate equitability?		
	What is your perspective of WIPAHS adopting projects aligned to the aims of the institute on to the projects portfolio? And how do you think this could be mobilised?		Adopt and affiliate like DECIPHER.

	What would be the potential benefits and weaknesses to WIPAHS and the researcher(s)?		Wider reach and support?<br>More paperwork?
Knowledge exchange	How can WIPAHS maximise on knowledge exchange?		
Impact	How do you think the work of WIPAHS can be translated to have an impact on health outcomes in Wales?	How do we measure that physical activity and health outcomes have improved as a result of the work of WIPAHS?	
**[Transition statement]**<br><br>Finally, considering future visions...			
**Future**			
Future	If you had three wishes for WIPAHS, what would they be?		Setting<br>Population<br>Interventions<br>Collaborations<br>Practices<br>Impact<br>Set up / processes
**Closing**			
That is all the questions I have for you today. The information you have been shared will be used to further develop WIPAHS. Before we finish, is there anything else you think would be helpful for me to know? Do you have any questions for me?<br><br>Thank you very much for you time and attention today. We appreciate you sharing your thoughts and time with us. Our contact details are on the consent form if you have any questions or concerns after today.			

**Prompts/Probes are there to be used as a guide for the interviewer. They are key words/phases to help the researcher ask questions and elicit responses from the participant. Prompting questions will be used in a conversational manner and only when deemed appropriate.*
