# Supplementary material for: The implementation and refinement of a national institute for physical activity, health and sport
Source: BMC Public Health. 2026 Mar 14;26:1313. doi: 10.1186/s12889-026-26524-z (PMC13101313; doi:10.1186/s12889-026-26524-z)
Supplement: Supplementary file 3 — Supplementary Material 3. [file 12889_2026_26524_MOESM3_ESM.pdf]

Sefydliad Cymru
Gweithgaredd Corfforol, Iechyd a Chwaraeon

Welsh Institute of Physical Activity, Health and Sport

Sit Less. Be Active. Play More.

A Brief Report on Healthcare Professionals' Approaches to Conversations Regarding the Promotion of Physical Activity and Sedentary Behaviour with Clinical Paediatric Populations in South Wales, UK

Report produced by:
Dr Rachel Knight, Dr Amie Richards, Prof Kelly Mackintosh, Dr Sarah Denford, Dr Callum Leese, Prof Joanne Hudson, Prof Gareth Stratton and Prof Melitta McNarry on *behalf of the Welsh Institute of Physical Activity, Health and Sport (WIPAHS)*

Authors in order of contribution with the last author being the Principal Investigator

August 2024

Table of Contents

Executive Summary ... 3

Introduction ... 5

Background ... 5

Scope of the Research ... 8

Process of the Study ... 9

Participants and Procedures ... 9

Results ... 9

Discussion and Next Steps ... 13

Recommendations ... 15

Recommendation 1 ... 15

Recommendation 2 ... 15

Recommendation 3 ... 16

References ... 17

Appendix ... 18

Appendix 1: Survey Questionnaire ... 18

Appendix 2: Data Analyses Processes ... 24

Descriptive Statisitics ... 24

Qualitative Content Analysis Process ... 24

Appendix 3: Additional Data Breakdown ... 25

Appendix 4: Report Strengths and Limitations ... 27

Executive Summary

Introduction

- Global levels of physical activity among children are low (Guthold et al., 2019). The latest Active Health Kids Report Card reported an 'F' for physical activity in Wales (Richards et al., 2021).
- Although the healthcare environment is one of the 'seven best investments' for physical activity promotion (GAPA, 2011), and the importance of physical activity conversations is recognised, approaches in such settings are informal and inconsistent (Lowe et al., 2022).

Method

- A 32-item questionnaire, adapted from Denford et al. (2020) was delivered via the JISC Online Survey Platform between 8th of June and 31st December 2023.
- The questionnaire explored clinicians' current practices in promoting physical activity and exercise, and reducing sedentary behaviour.

Results

- 100% of participants personally felt the topic of physical activity was important or very important in their patients healthcare, with 93% placing the same importance to the topics of exercise and sedentary behaviour.
- Only 63% of respondents felt the topic of physical activity was adequately discussed, with this figure reducing to 56% for exercise, and 49% for sedentary behaviour.

Conclusion

- Most participants highly valued discussing physical activity, exercise, and sedentary behaviour; feeling confident and competent. However, where no advice was provided, or difficulties arised doing so in different environments, lacking knowledge and confidence was a key contributor.
- Overall, resource availability (time, capacity, or space) was the primary barrier, whereas knowledge, confidence, resources, and access to community-led support and provision were identified as key facilitators.

Recommendation 1

- **Communication of core messages** that include the use of the right terminology in the **right way**, at the **right time**, by the **right person,** for the paediatric patient and their family.

Recommendation 2

- Engage in further work to **explore the specific roles of different multi-disciplinary team members** across primary and secondary care regarding discussions about physical activity, exercise, and sedentary behaviour, the barriers they face, and key facilitators.

Recommendation 3

- Create a **toolkit to support clinicians** to promote physical activity and exercise, and reductions in sedentary behaviour to children, young people, and their families.

Introduction

Following the Welsh Institute of Physical Activity, Health and Sport (WIPAHS) Roadshow in 2022, Dr Nick Wilkinson, a Consultant Paediatrician from Cardiff and Vale University Health Board (CAVUHB) and also Officer for Wales at Royal College of Paediatrics and Child Health, identified an insight gap regarding the barriers and facilitators to Wales-based Healthcare Professionals (HCPs) engaging in discussions around physical activity, exercise and sedentary behaviour with their paediatric patients and their families. WIPAHS worked alongside CAVUHB to address this gap by undertaking a survey of current practices.

Background

The importance of being physically active (including exercise participation), and limiting sedentary behaviour for health and well-being are well recognised, irrespective of age or the presence of any underlying health condition(s) (Marquez et al., 2020). Indeed, this is reflected in the integration of physical activity and sedentary behaviour management as a cornerstone of many healthcare-based treatment and intervention strategies. Nonetheless, this remains an area of misunderstanding and poor implementation for many clinically-led services. It is pivotal to understand misconceptions, barriers and facilitators to promoting physical activity and exercise, and reducing sedentary behaviours, from the perspectives of HCP's working with clinical populations. Developing this knowledge and understanding will enable effective informing of future practice and service delivery.

Box 1. Key Definitions

Physical Activity:
Any bodily movement produced by skeletal muscles that results in energy expenditure that requires energy to be used (above resting; Caspersen et al., 1985).

Exercise:
Physical activity that is planned, structured, repetitive, and purposive in the sense that improvement or maintenance of one or more components of physical fitness is an objective (Caspersen et al., 1985).

Sedentary Behaviour:
Doing very little movement whilst being in a lying or sitting position during waking hours and using not much more energy than that used at rest. This is characterised by an energy expenditure $\leq$ 1.5 metabolic equivalents (METs; Tremblay et al., 2017).

Healthcare Professional:
Any individual employed in a healthcare role (any discipline), involved in the treatment of at least one paediatric population.

Why promote physical activity?

Figures 1-3 show infographics from the 2019 United Kingdom's Chief Medical Officers' updated physical activity guidelines outlining the numerous benefits for children and young people of being physically active. Such benefits are achievable through a range of opportunities, from structured attendance at organised sports, to engaging with activities in the local community, to purely engaging in physical play. Importantly, these activities can, and need to be, incorporated into children's daily lives to help them optimise their health.

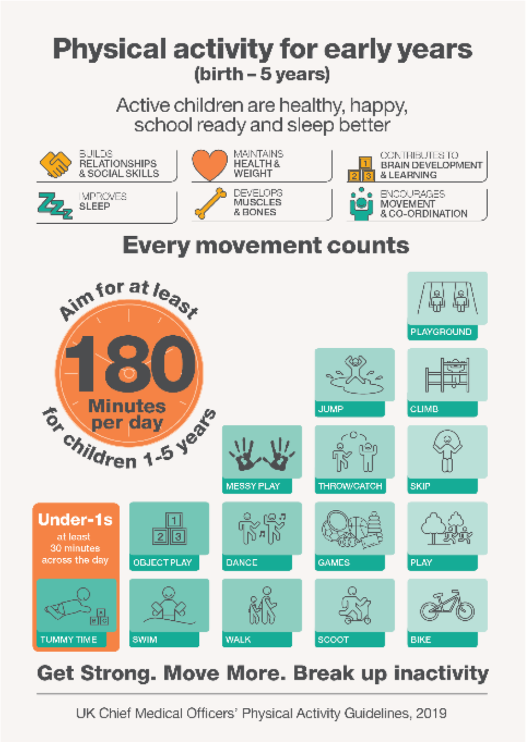

Figure 1. Physical activity guidelines for early years

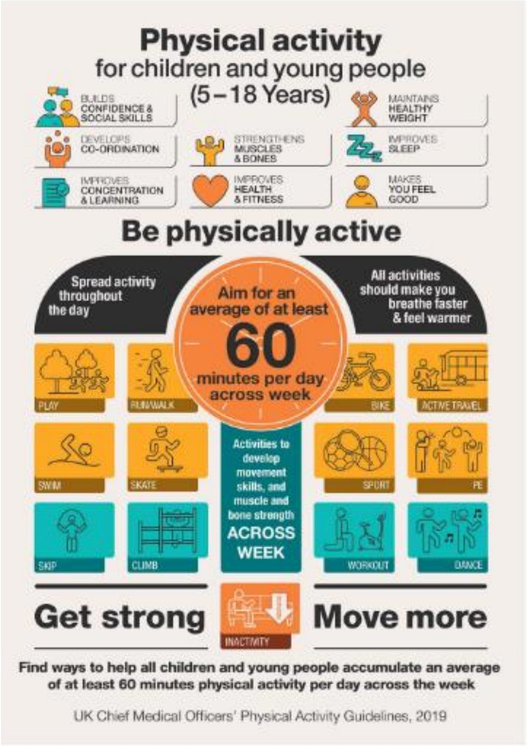

Figure 2. Physical activity guidelines for 5 - 18 years

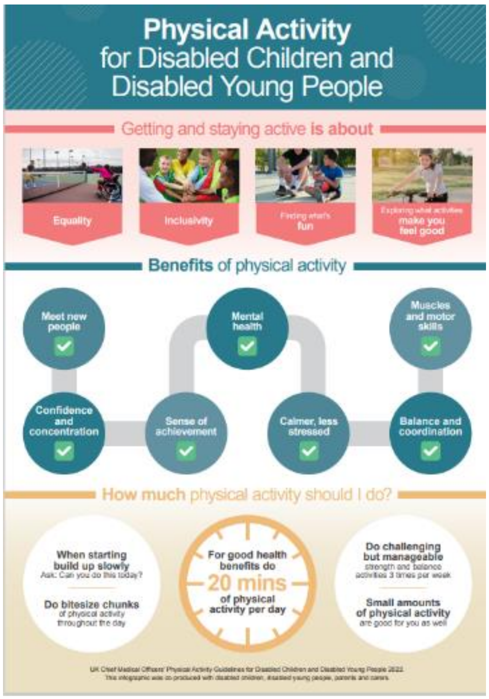

Figure 3. Physical activity guidelines for disabled children and disabled young people

Worldwide, children and young people's physical activity levels are low and have decreased over time (Guthold et al., 2019). This is no different in Wales, and was further exacerbated by the COVID-19 pandemic (Hurter et al., 2022). The latest Active Health Kids Report Card, scoring Wales as an 'F' for physical activity (Richards et al., 2022); the lowest of grade attainable. The report card results, in correlation with global trends (Guthold et al., 2020), highlight that only 17% of young people were active for 60 minutes per day, with this decreasing to 14% when only considering moderate-to-vigorous physical activity. Furthermore, considerable inequalities were reported in relation to sex, socio-economic status and ethnicity (Page et al., 2021). Moreover, from the eleven indicators that were graded within the Active Healthy Kids Report Card, only one provided any data on children with disabilities. The organised sport indicator, from the Sport Wales School Sport Survey, found that 33% of students with an impairment or disability participated in sport three or more times per week compared with 45% of those without a disability or impairment; this gap has widened since the last report card (Richards et al., 2022).

Why Target HCPs to Promote Physical Activity and What Do We Already Know?

The healthcare environment is seen to be one of the 'seven best investments' for physical activity promotion (GAPA, 2011). HCPs are deemed to be valued, trusted, and a credible source of physical activity information (Macmillan, 2017). However, despite evidence that conversations about physical activity are perceived as important (i.e. by 99% of 839 UK General Practitioners surveyed; Lowe et al., 2022) and brief interventions are regularly delivered ; Lowe et al., 2018) approaches to physical activity promotion in healthcare settings are often informal and inconsistent. Formal frameworks are rarely used, with brief interventions poorly understood and integrated (Lowe, Littlewood and Mclean, 2018), and a range of barriers to promotion having been identified – see Figure 4 (Lobello at al., 2020; Lowe, Littlewood & Mclean, 2018; Lowe et al., 2022; Netherway, Smith & Monforte, 2021).

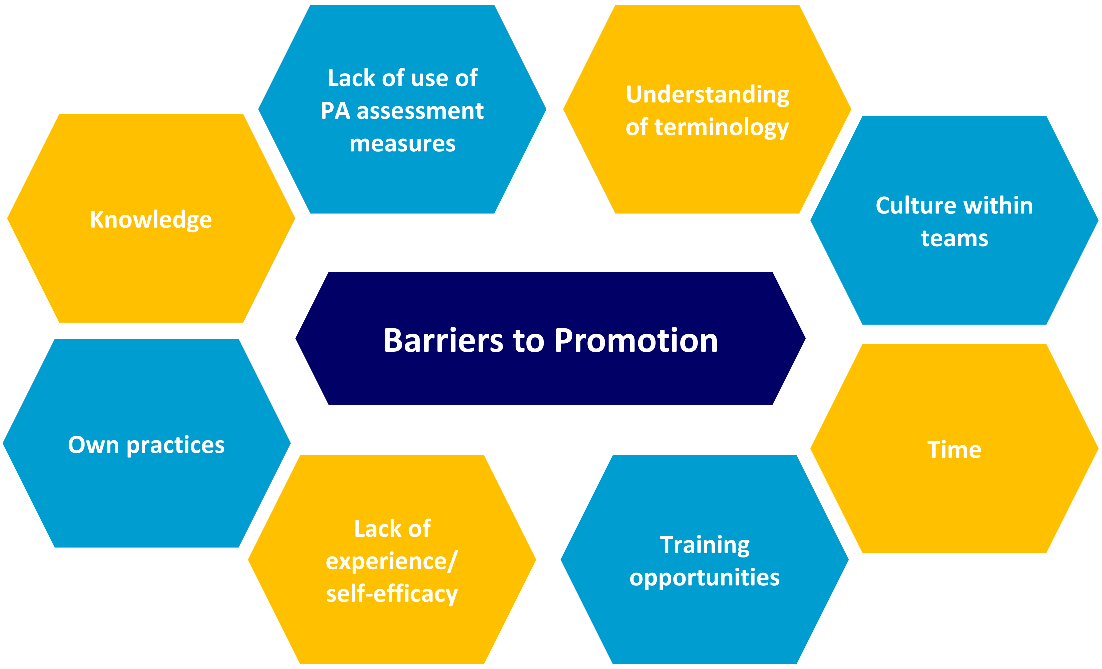

Figure 4. Barriers to physical activity promotion by HCPs (Lobello at al., 2020; Lowe, Littlewood and Mclean, 2018; Lowe et al., 2022; Netherway et al., 2021)

Scope of the Research

Dr Nick Wilkinson (Consultant Paediatrician, CAVUHB) expressed an interest in collaborating with WIPAHS to explore topics relating to physical activity, exercise and sedentary behaviours in paediatric patient populations across Wales. Following consultation, a workshop aiming to spark conversations about how we encourage physical activity in clinical paediatric populations, and provide opportunities for networking and shared learning regarding the methods currently used across the seven Welsh Health Boards, was held at Swansea University Bay Campus (attended by 24 HCPs). To ensure that the insights gained from this work were generalisable across Wales, and maximise their impact, we subsequently developed and implemented a nationwide (United Kingdom; UK) survey to gain further perspectives on the promotion of physical activity and exercise, and sedentary behaviour reduction in such populations.

Box 2 outlines the research questions which were addressed in the survey and underpin this report.

Box 2. Research questions explored in this report:

1. What are HCPs' current practices regarding the promotion of physical activity and exercise, and the reduction of sedentary behaviour, with clinical paediatric populations (aged 0-18 years)?

2. What barriers do HCPs encounter when having conversations about and providing opportunites to enhance physical activity and exercise, and reduce sedentary behaviour, with children, young people, and their families?

3. What terminology and language do HCPs use when engaging with children, young people, and their families about physical activity, exercise, and sedentary behaviour?

Process of the Study

Participants and Procedures

A single 32-item questionnaire, adapted from the work of Denford et al. (2020), was delivered via the JISC Online Survey Platform between the 8th June 2023 and 31st December 2023. The questionnaire, available in Appendix 1, was designed by the research team to explore clinicians' current practices centred around physical activity and exercise promotion, and sedentary behaviour reduction. For descriptive purposes, basic demographic data, namely, gender, age, profession, and work/clinic location was also captured.

All participants provided electronic informed consent prior to survey completion. Ethics approval was granted by Swansea University Research Ethics Committee (approval number: 1 2023 6688 5708).

To be eligible to participate, individuals must have been:

⇒ Employed in a healthcare role (any discipline);
⇒ Involved in the treatment of at least one paediatric population; and,
⇒ Able to converse fluently in English.

Participants were recruited from attendees of the workshop held at Swansea University, and via pre-existing professional networks and social media.

The survey data obtained were analysed using a mixture of **descriptive statistics and the principles of Qualitative Content Analysis** (see Graneheim & Lundman, 2004, Hsieh & Shannon, 2005). Further details of the data analysis processes are provided in Appendix 2. Direct quotations are presented in italics.

Results

The survey was completed by 47 participants across the UK. As a result of low response rates from participants who resided outside of South Wales (n = 6), this data was excluded, resulting in data from **41 participants from South Wales being included in the final analysis**. Demographic details relating to the *participants*; their self-perceived *proficiency* regarding understanding of, and ability to, discuss key terms; their *perspectives* on the personal importance attached to discussing topics and how well they are addressed within their services; and, current *practices* around discussions held about physical activity, exercise, and sedentary behaviour are presented in a pen profile (Figure 5).

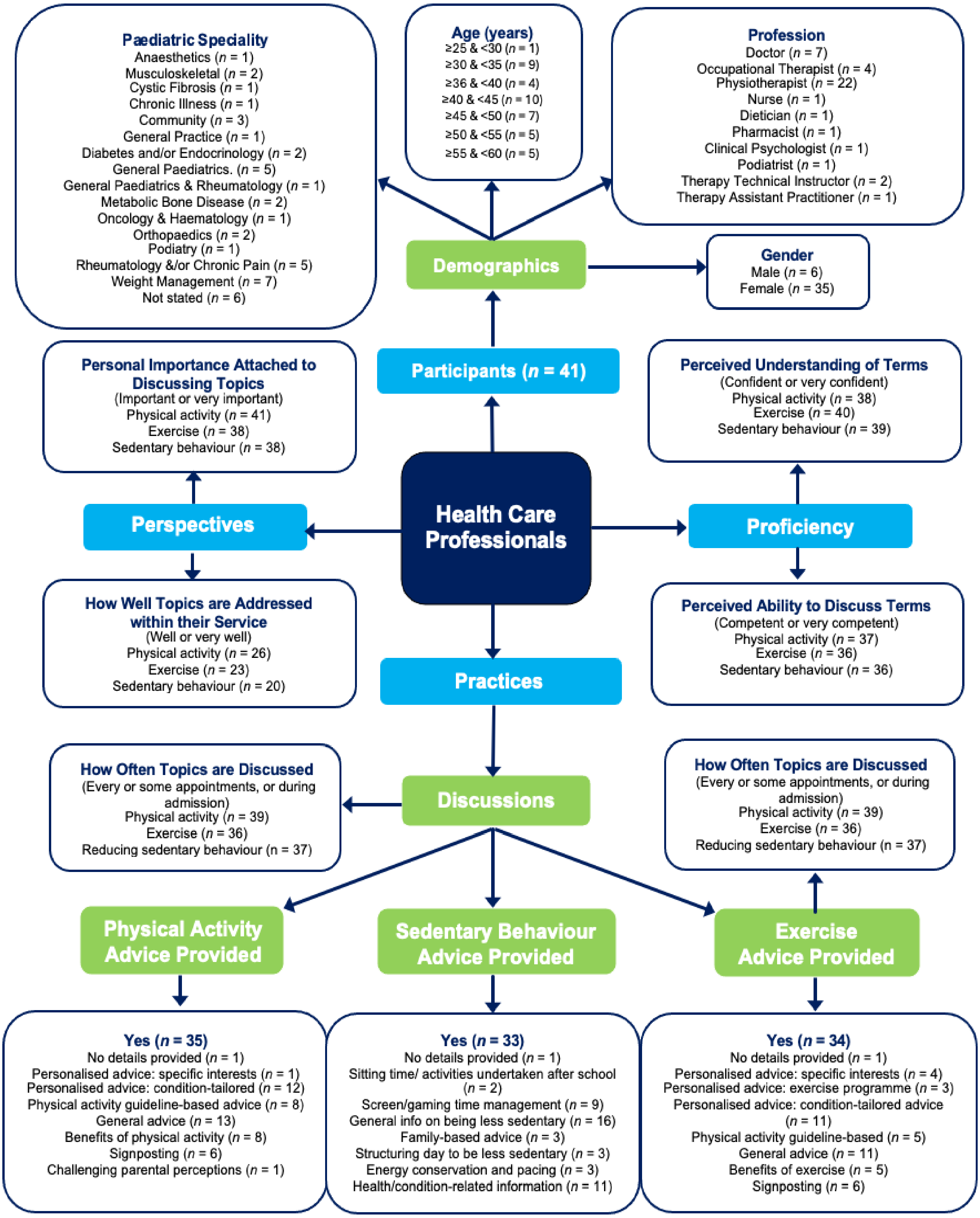

Figure 5. Pen profile of key participant demographics, perceived proficiency, perspectives, and current practices relating to discussions about physical activity, exercise and sedentary behaviour.

Within the pen profile, it is highlighted that whilst **100% of participants personally felt the topic physical activity was important or very important** in their patients healthcare, and only slightly less attached the same importance level to the topics exercise and sedentary behaviour (93%), opinions on how well topics were addressed within services varied. **Only 63% of respondents felt the topic of physical activity was adequately discussed, with this figure only 56% for exercise, and 49% for sedentary behaviour**.

The topic of physical activity was reported to be discussed at every or some appointments, or during patient admissions, by 95% of respondents (39/41):

> "*Depending on how frequently I see [the] patient, I may not discuss at each appointment, but will always discuss with every patient*" (Participant 3).

Indicating physical activity was only discussed in 'some appointments' related to a variety of reasons from the type of clinical condition, whether the patient is new to caseload or long term, frequency of appointments and preferring "*conversations being elicited by the family*" (Participant 14).

Patterns of discussion were similar for the topic of exercise (88%; 36/41 discussed at every or some appointments or always during patient admission). Reasons for not discussing exercise in every appointment mirrored those for physical activity, with the addition of inferences towards conversations needing to be "*relevant to the patient at that time, at that age*" (Participant 39) and "*beneficial*" (Participant 14) within the wider conversation.

Regarding sedentary behaviour, again responses were similar (90%; 37/41 discussed at every or some appointments or always during patient admission), however one respondent did report never having this discussion and another stated that for this topic "*conversations are difficult*" (Participant 10).[1]

Four common themes were identified across topics when no advice was given:

- ⇒ **Time/resources** (physical activity 2/6; exercise 2/7; sedentary behaviour 3/8)
- ⇒ A **lack of knowledge or confidence** in what advice to give (physical activity 2/6; exercise 2/7; sedentary behaviour 3/8)
- ⇒ **Other priorities** (physical activity 3/6; exercise 4/7; sedentary behaviour 3/8)
- ⇒ Being **led by the patient/families** (physical activity 3/6; exercise 3/7; sedentary behaviour 3/8)

[1] N.B For each theme, the numbers indicate the number of respondents who indicated that reason for not giving advice/ the total number of respondents who didn't give advice on the topic i.e. for Time/resources, physical activity = 2/6

For participants who stated that advice on a particular topic was provided, a breakdown of which areas are regularly discussed with children, young people and their families is outlined in Figure 6.

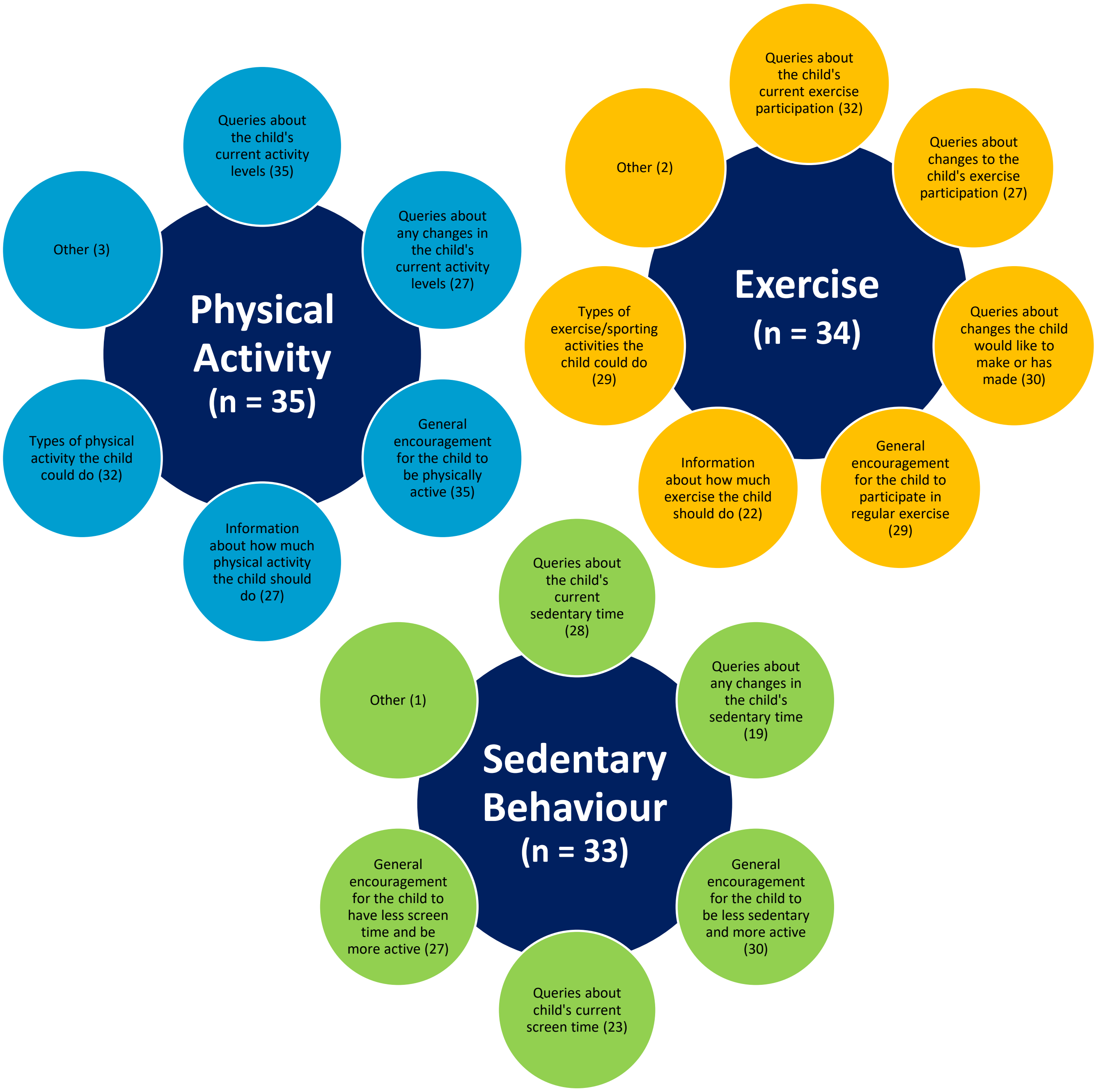

Figure 6: Specific areas discussed regularly during consultations (from multiple choice options; all that apply were chosen)[2]

[2] N.B For each response, the number in parenthesis indicates the number of respondants who ticked that option out of the total number of respondants who stated yes, they regularly ask questions and/or provide advice on each topic i.e. for physical activity 'other' = 3/35.

Prescribed exercise programmes were reported to be offered within **only 56% of the clinical areas represented.**

Resource availability (including time and capacity; 8/23) and **preferring to focus on physical activity and/or general function** (5/23) were presented as key reasons for exercise-programme prescription not occurring. Additional **space/facilities** (13/23), **resources** (staff/funding/time; 13/23), and **support from/opportunities within the community** (10/23) were the most commonly identified factors that would facilitate the ability to provide this option within their service.

Similar factors were raised to improve ability to facilitate physical activity and/or exercise at a patient's home, with **increasing knowledge** (11/23) also deemed important. Along with increased knowledge, to promote reductions in sedentary behaviour both at their facility and within patients' homes, clinicians would also value having **access to improved information/tools** (12/23 and 10/23 respectively). Perceived reasons for patients regularly (at least once a week) being prevented from being physically active varied; full breakdown of all responses is provided in Appendix 3, along with additional information on exercise prescription and promotion.

Discussion and Next Steps

This report provides insight into HCPs perspectives of physical activity, exercise and sedentary behaviour conversations and promotion with clinical paediatric populations and their families in South Wales, UK. The importance placed on discussing the topics of physical activity, exercise, and sedentary behaviour, and the confidence and competence to do so, was perceived to be high by HCP respondants in this study. However, how well these are addressed within services and across clinical practice varies, and when no advice was provided, or limitations were identified providing advice in different environments (i.e. a patients home) a lack of, or needing to increase knowledge was a key driving factor.

The **terminology** used within conversations could have significant implications on the messages delivered. The findings reported here indicate that in South Wales, there is disparity in the understanding and application of core terminology, particularly regarding the term '*exercise*'. Where exercise is defined as: physical activity that is planned, structured, repetitive, and purposive in the sense that improvement or maintenance of one or more components of physical fitness is an objective (Caspersen et al., 1985), it can sometimes be misaligned with the provision of '*graded exercise programmes*' prescribed to target a specific condition or musculoskeletal problem. Previous research by Lowe et al. (2018) found that a

lack of clarity was also situated around which concepts were promoted; with the wider benefits of physical activity for long-term health and wellbeing often being overlooked in favour of focusing on targeted exercise for short-term fitness-based gains.

Inferences about whose responsibility it was deemed to be to have conversations could not be drawn. However, the **current practices** of this respondant group revealed that who has discussions, depends on: i) who is part of the multidisciplinary team (MDT); and ii) whether there is an MDT, versus just an individual practitioner. Additionally, some HCPs believed the most effective strategy was to be led by when families inititated discussions. Overall, it is alluded that **responsibility does not lie with only one profession**, and that, within MDTs, it is becoming, if indeed it is not already, the responsibility of everyone who has contact with the patient, regardless of clinical role or speciality. Where MDT approaches are insitu, continuity of communication across the team, and the provision of consist messages will be paramount.

Similar to previous research findings (Lobello at al., 2020; Lowe, Littlewood & Mclean, 2018; Lowe et al., 2022; Netherway, Smith & Monforte, 2021), **barriers** to being able to have conversations and/or deliver opportunites for prescribed exercise programmes were primarily limited by **resource availability** (albeit time, capacity or space). **Increasing knowledge and information, resources and access to community-led support and provision** were highlighted as **key facilitators**. Intitatives like the National Exercise Referal Scheme (NERS)[3], Good Boost[4], and the Health Disability Activity Pathway (HDAP)[5], are available to assist HCPs across Wales to optimise discussions and the promotion of opportunities to be physically active. Where NERS and Good Boost are only available to individuals aged 16+ years, the HDAP is, locality offerings dependent, accessible to all from aged 2+ years. With a focus on increasing the number of disabled people participating in physical activity (including sport), the pathway, a collaboration between health, Disability Sport Wales and Local Authorities, provides HCPs with a structured approach to refer individuals for signposting to inclusive opportunites within the community.

However, it is plausible that uptake to, and enagement with, opportunities is directly related to the quality of the discussions held with families at the point of referral. For example, whilst the HDAP process provides an element of HCP education it is apparent that to comprehensively challenge perceptions and optimise discussion effectiveness further integration of training opportunities is required. Greater **integration of education components centred around physical activity (including exercise) promotion, and reducing sedentary behaviour, within HCP pre-registration courses**, could be one avenue to embed such practices.

[3] National Exercise Referral Scheme: https://phw.nhs.wales/services-and-teams/wales-national-exercise-referral-scheme/
[4] Good Boost: https://www.goodboost.ai/ Access dependant on local health board and service provision
[5] Health Disability Activity Pathway: https://www.hdapathway.co.uk/

Recommendations

Facilitating conversations with clinical paediatric populations and their families can be challenging. Based on the findings of this report, **three key recommendations** have been made. Whilst these provide key areas for practitioner, service delivery, and policy-maker consideration, some also require more work to explore and further understand how to optimise their impact.

Recommendation 1

Communication of core messages that include the use of the correct terminology in the right way, at the right time, by the person deemed to be the right person for the paediatric patient and their family.

Clear consistent communication: **Between MDT members to allow for continuity of messaging**.

Clear consistent communication: **To clinical paediatric populations and their families** to optimise engagement with opportinuties to be physically active.

Recommendation 2

Engage in further work to explore the specific roles of different MDT members across primary and secondary care regarding discussions about physical activity, the barriers they face, and key facilitators.

Across primary and secondary care, identify the **specific barriers different MDTs and MDT-members face** when trying to engage in discussions about physical activity, exercise, and sedentary behaviour, with clinical paediatric populations and their families.

Establish what key facilitators and opportunities need to be available to support HCPs in different roles and clinical settings.

Recommendation 3

Create a toolkit to support clinicians to promote physical activity and exercise, and reductions in sedentary behaviour to children, young people and their families.

Develop a menu-based approach that contains a **series of optimised easily accessible options, strategies and resources**: consideration should be given to options such as the use of gamification in healthcare settings, online resources, intergenerational/ family-based activities, and facilitating engagement with community-based opportunities.

References

Campbell, A., Foster, J., Stevinson, C., & Cavill, N. (2017). The importance of physical activity for people living with and beyond cancer. A concise evidence review. *Macmillan Cancer Support.* Available at: *https://be.macmillan.org.uk/Downloads/CancerInformation/LivingWithAndAfterCancer/MAC138200415PhysicalActivityevidencereviewDIGITAL.pdf [Accessed 13 August 2024].*

Caspersen, C. J., Powell, K. E., & Christenson, G. M. (1985). Physical activity, exercise and physical fitness: definitions and distinctions for health-related research. *Public Health Rep*, 100, 126–131.

Denford, S., Cox, N.S., Makintosh, K.A., McNarry, M., O'Halloran, P., Holland, A. E., Tomlinson, O.W., Barker, A. R., and Williams, C.A. (2020). Physical activity for cystic fibrosis: perceptions of people with cystic fibrosis, parents and healthcare professionals. *ERJ Open Research*, 6(3). http://doi.org/10.1183/23120541.00294-2019

Global Advocacy for Physical Activity IS for PA and H. Investments that Work for Physical Activity. Published Online First: 2011. https://www.globalpa.org.uk/pdf/investments-work.pdf (accessed 19 July 2024).

Guthold, R., Stevens, G.A., Riley, L.M., and Bull, F.C. (2020). Global trends in insufficient physical activity among adolescents: a pooled analysis of 298 population-based surveys with 1· 6 million participants. *The Lancet Child & Adolescent Health* 4(1): 23-35. https://doi.org/10.1016/S2352-4642(19)30323-2

Hurter, L., McNarry, M.A., Stratton, G., & Mackintosh, K.A. (2022). Back to school after lockdown: The effect of COVID-19 restrictions on children's device-based physical activity metrics. Journal of Sport and Health Science, 11(4). https://doi.org/10.1016/j.jshs.2022.01.009

Kohl, H. W., Cook, H. D., Van Dusen, D. P., Kelder, S. H., Ranjit, N., & Perry, C. L. (2013). Educating the study body: taking physical activity and physical education to school. Chapter 4: Physical Activity, Fitness, and Physical Education: Effects on Academic Performance. *Journal of School Health*, 81(12).

Lobelo, F., Muth, N.D., Hanson, S., and Nemeth, B.A. (2020) Physical Activity Assessment and Counseling in Pediatric Clinical Settings. *Pediatrics*, 145(3):e20193992. http://doi.org/10.1542/peds.2019-3992

Lowe, A., Littlewood, C., & Mclean, S. (2018). Understanding physical activity promotion in physiotherapy practice: A qualitative study. *Musculoskeletal Science and Practice*, 35, 1-7. http://doi.org/10.1016/j.msksp.2018.01.009

Lowe, A., Myers, A., Quirk, H., Blackshaw, J., Palanee, S., & Copeland, R. (2022). Physical activity promotion by GPs: a cross-sectional survey in England. *BJGP Open.* http://doi.org/10.3399/BJGPO.2021.0227

Marquez DX, Aguiñaga S, Vásquez PM, Conroy DE, Erickson KI, Hillman C, Stillman CM, Ballard RM, Sheppard BB, Petruzzello SJ, King AC, Powell KE. (2020). A systematic review of physical activity and quality of life and well-being. *Translational Behavioural Medicine,* 10(5), 1098-1109. https://doi.org/10.1093/tbm/ibz198.

Netherway, J., Smith, B., and Monforte, J. (2021). Training Healthcare Professionals on How to Promote Physical Activity in the UK: A Scoping Review of Current Trends and Future Opportunities. *International Journal of Environmental Research and Public Heath*, 18(13), 6701. https://doi.org/10.3390/ijerph18136701

Page, N., Hewitt, G., Young, H., Moore, G., & Murphy, S. (2021). Student Health and Wellbeing in Wales: Report of the 2019/20 School Health Research Network Student Health and Wellbeing Survey.

Richards, A. B., Mackintosh, K. A., Swindell, N., Ward, M., Marchant, E., James, M., Edwards, L. C., Tyler, R., Blain, D., Wainwright, N., Nicholls, S., Mannello, M., Morgan, K., Evans, T., & Stratton, G. (2022). WALES 2021 Active Healthy Kids (AHK) Report Card: The Fourth Pandemic of Childhood Inactivity. *International Journal of Environmental Research and Public Health*, 19(13). https://doi.org/10.3390/ijerph19138138

Tremblay, M. S., Aubert, S., Barnes, J. D., Saunders, T. J., Carson, V., LatimerCheung, A. E., Chastin, S. F. M., Altenburg, T. M., Chinapaw, M. J. M., Aminian, S., Arundell, L., Hinkley, T., Hnatiuk, J., Atkin, A. J., Belanger, K., Chaput, J. P., Gunnell, K., Larouche, R., Manyanga, T., … Wondergem, R. (2017). Sedentary Behavior Research Network (SBRN) - Terminology Consensus Project process and outcome. *International Journal of Behavioral Nutrition and Physical Activity*, 14(1). https://doi.org/10.1186/s12966-017-0525-8

Appendix

Appendix 1: Survey Questionnaire

a) About you

1. **Gender**

Male	[ ]
Transgender male	[ ]
Female	[ ]
Transgender female	[ ]
Non-Binary	[ ]
Prefer not to say	[ ]

2. **Age** _______________

b) The clinic

1. Locality of clinic: ____
2. Speciality of clinic: ____
3. Your position (please circle): Doctor / Physiotherapist / Occupational therapist/ other (please specify): ____
4. How many children and adolescents are registered in your clinic? ______________________

c) Physical activity, exercise, and sedentary behaviour

1. Using a scale of 1 (not very confident) to 5 (very confident), indicate how confident you are in your understanding of each of the following terms (please circle).

Physical activity

1	2	3	4	5
(not very confident)		(neutral)		(very confident)

Exercise

1	2	3	4	5
(not very confident)		(neutral)		(very confident)

Sedentary behaviour

1	2	3	4	5
(not very confident)		(neutral)		(very confident)

2. Using a scale of 1 (not very competent) to 5 (very competent), indicate how competent you feel to discuss each of the following with your patients (please circle).

Physical activity

1	2	3	4	5
(not very confident)		(neutral)		(very confident)

Exercise

1	2	3	4	5
(not very confident)		(neutral)		(very confident)

Reducing sedentary behaviour

1	2	3	4	5
(not very confident)		(neutral)		(very confident)

Please review the following definitions and consider these when answering the questions that follow:

Physical activity: “Any bodily movement produced by skeletal muscles that results in energy expenditure”. (Caspersen et al., 1985).

Exercise: “Physical activity that is planned, structured, repetitive, and purposive in the sense that improvement or maintenance of one or more components of physical fitness is an objective”. (Caspersen et al., 1985)

Sedentary Behaviour: “Any waking behavior characterized by an energy expenditure ≤1.5 metabolic equivalents (METs), while in a sitting, reclining or lying posture”. (Tremblay et al., 2017)

3. Using a scale of 1 (not very confident) to 5 (very confident), indicate how confident you are in your understanding of each of the following terms (please circle).

Physical activity

1	2	3	4	5
(not very confident)		(neutral)		(very confident)

Exercise

1	2	3	4	5
(not very confident)		(neutral)		(very confident)

Sedentary behaviour

1	2	3	4	5
(not very confident)		(neutral)		(very confident)

4. Using a scale of 1 (not very competent) to 5 (very competent), indicate how competent you feel to discuss each of the following with your patients (please circle).

Physical activity

1	2	3	4	5
(not very confident)		(neutral)		(very confident)

Exercise

1	2	3	4	5
(not very confident)		(neutral)		(very confident)

Reducing sedentary behaviour

1	2	3	4	5
(not very confident)		(neutral)		(very confident)

5. Using a scale of 1 (not important at all) to 5 (very important), indicate the importance that you personally attach to each of the following topics in the healthcare of the patient (please circle).

Physical activity

1	2	3	4	5
(not important at all)		(neutral)		(very important)

Exercise

1	2	3	4	5
(not important at all)		(neutral)		(very important)

Reducing sedentary behaviour

1	2	3	4	5
(not important at all)		(neutral)		(very important)

6. Using a scale of 1 (not at all) to 5 (very well), how well do you feel each of the following topics are addressed within your service (please circle)?

Physical activity

1	2	3	4	5
(not at all)		(neutral)		(very well)

Exercise

1	2	3	4	5
(not at all)		(neutral)		(very well)

Reducing sedentary behaviour

1	2	3	4	5
(not at all)		(neutral)		(very well)

7. If at all, how often do you discuss the following with your patients (please tick as many as appropriate)?

Physical activity

Never [ ]
Once a year [ ]
Only if mentioned by the patient [ ]
At every appointment [ ]
At some appointments [ ]
During inpatient admission [ ]
Other [ ] please specify____
If more than one box ticked, please explain:____

Exercise

Never [ ]
Once a year [ ]
When a patient reports exercise difficulties [ ]
Only if mentioned by the patient [ ]
At every appointment [ ]
At some appointments [ ]
During inpatient admission [ ]
Other [ ] please specify__________________________________
If more than one box ticked, please explain:____

Reducing sedentary behaviour

Never [ ]
Once a year [ ]
When a patient reports exercise difficulties [ ]
Only if mentioned by the patient [ ]
At every appointment [ ]
At some appointments [ ]
During inpatient admission [ ]
Other [ ] please specify__________________________________
If more than one box ticked, please explain: ____

---

8. In the clinic, whose responsibility is it to discuss each of the following topics with the patient (please tick as many as appropriate)?

Physical activity

Doctor	[ ]	Therapy assistant	[ ]
Nurse	[ ]	Health care support worker	[ ]
Physiotherapist	[ ]	Exercise professional	[ ]
Don't know	[ ]		

Other personal please specify: ____

Exercise

Doctor	[ ]	Therapy assistant	[ ]
Nurse	[ ]	Health care support worker	[ ]
Physiotherapist	[ ]	Exercise professional	[ ]
Don't know	[ ]		

Other personal please specify: ____

Reducing sedentary behaviour

Doctor	[ ]	Therapy assistant	[ ]
Nurse	[ ]	Health care support worker	[ ]
Physiotherapist	[ ]	Exercise professional	[ ]
Don't know	[ ]		

Other personal please specify: ____

9. Is advice given to patients on the topic of physical activity?

Yes [ ]
No [ ]

10. If yes, what advice is given
If no, please explain why not____

11. If yes, which of these specific topics are discussed (tick all that apply)

Queries about the child's current activity levels ☐
Queries about any changes in the child's current activity levels ☐
General encouragement for the child to be physically active ☐
Information about how much physical activity the child should do ☐
Types of physical activity the child could do ☐
Other ☐ (please specify) ____

12. Is advice given to patients on the topic of exercise?

Yes [ ]
No [ ]

13. If yes, what advice is given
If no, please explain why not____

14. If yes, which of these specific topics are discussed (tick all that apply)

Queries about the child's current exercise participation ☐
Queries about any changes in the child's exercise participation ☐
Queries about changes the child would like to make or have made ☐
General encouragement for the child to participate in regular exercise ☐
Information about how much exercise the child should do ☐
Types of exercise/sporting activities the child could do ☐
Other ☐ (please specify) ____

15. Is advice given to patients on the topic of sedentary behaviour?

Yes [ ]
No [ ]

16. If yes, what advice is given
If no, please explain why not ____

17. If yes, which of these specific topics are discussed

Queries about the child's current sedentary time ☐
Queries about any changes in the child's sedentary time ☐
General encouragement for the child to be less sedentary and more active ☐
Queries about child's current screen time ☐
General encouragement for the child to have less screen time and be more active ☐
Other ☐ (please specify)____

18. Does the clinic offer a prescribed exercise training programme for patients (please tick)?

Don't know [ ]
Yes [ ]
No [ ]

19. If yes, please describe ____
20. If no, please explain why (e.g., timing, resources, finances, lack of need etc ______________________
21. What would improve your ability to facilitate exercise to be undertaken within the facility by patients? ____
22. What would improve your ability to promote and facilitate exercise and/or physical activity for your patients at home? ____
23. What would improve your ability to promote reductions in sedentary behaviour within the facility for your patients? ____
24. What would improve your ability to promote reductions in sedentary behaviour for your patients at home? ____

25. What do you perceive to be the main reasons your patients are regularly (e.g., at least once a week) **prevented** from being physically active (through exercise or physical activity)

Lack of time ☐
Lack of enjoyment of activity ☐
Tiredness ☐
Unwell ☐
School/homework pressure ☐
Peer pressure ☐
Would rather do something else with spare time ☐
Concerns about appearance ☐

Concerns about exacerbating symptoms ☐
Concerns about the safety of physical activity with their condition ☐
Unclear what type / intensity of physical activity to do ☐
Family concerns ☐
Other (please state)____

26. What do you perceive to be the main factors that would **encourage** your patients (e.g., at least once a week) to be more physically active (through exercise or physical activity)

More time ☐
Enjoyment of activity ☐
Less School/homework pressure ☐
Peer support ☐
Provision of opportunities by your clinic ☐
Provision of opportunities with other children with the same or similar conditions ☐
Provision of information about managing symptoms during physical activity ☐
Education about the safety of physical activity with their condition ☐
Education about the benefits of physical activity with their condition ☐
Education about what type/ intensity of physical activity to do ☐
Family involvement ☐
Other (please state)____

Appendix 2: Data Analyses Processes

Descriptive Statisitics

Demographic data, and the responses to closed and Likert-Scale questions were analsysed using basic descriptive statistics. The limited number of responses received precluded the undertaking of a more detailed analysis and use of interferential techniques.

Qualitative Content Analysis Process

For free-text questions, taking a manifest approach, one author (RLK), for each individual question in isolation:

i) reviewed the responses, coding only the direct meaning of the data at a word or phrase level;

ii) grouped the identified codes together to form question response themes; and,

iii) conducted a frequency analysis of the generated themes to explore which approaches, techniques and terminology were used the most often and/or deemed the most useful.

Discussions were held with additional author (ABR) at key points throughout the process to review and discuss the codes and themes and provide critical interpretation.

Appendix 3: Additional Data Breakdown

Full breakdown of all perceived reason responses for patients regularly (at least once a week) being prevented from being physically active varied along with additional information on exercise prescription and promotion.

Question	Responses
Does the clinic offer a prescribed exercise training programme for patients (free-text responses)	Yes (n = 23)<br>Implied graded programme of exercises (11)<br>Implied exercise focused on fitness and health (6)<br>No answer/unclear (5)<br>(Referal to) external agency (1)<br><br>No (n = 18)<br>Resources/time/capacity to do (8)<br>Not appropriate (1)<br>Unsure why not (1)<br>Focus on physical activity and/or general function (5)<br>Don't know if offered (3)
What would improve your ability to facilitate exercise to be undertaken within the facility by patients? (free text responses)	Support from community led services/ supported referral/signposting options (10)<br>Online support (1)<br>Space/Facilities (13)<br>Extra staff/funding/time (13)<br>Not applicable in acute settings (3)<br>Increased knowledge (5)<br>Nothing/have what is needed (5)<br>Other (3)
What would improve your ability to promote and facilitate exercise and/or physical activity for your patients at home? (free text responses)	Support from community led services/ supported referral/signposting options (12)<br>Better Online support (4)<br>Space/Facilities (2)<br>Extra staff/funding/time (12)<br>Family engagement (5)<br>Increased knowledge (11)<br>Nothing/have what is needed (3)<br>Other (1)
What would improve your ability to promote reductions in sedentary behaviour within the facility for your patients? (free-text responses)	Unsure (1)<br>Improved information/tools (12)<br>Use of screen time reminders (1)<br>Community Support (5)<br>Staffing (2)<br>Knowledge (9)<br>Time (4)<br>Nothing/already do this (5)<br>Facilities/resources (4)<br>Other (2)

What would improve your ability to promote reductions in sedentary behaviour for your patients at home? (free-text responses)	Family engagement (8)<br>Unsure (1)<br>Improved information/tools (10)<br>Use of screen time reminders (1)<br>Community Support (6| Staffing (3)<br>Knowledge (11)<br>Time (5)<br>Nothing/already do this (2)<br>Facilities/resources (3)<br>Other (2)
What do you perceive to be the main reasons your patients are regularly (e.g. at least once a week) prevented from being physically active (through exercise or physical activity)? (multiple choice answers)	Lack of time (16)<br>Lack of enjoyment of activity (26)<br>Tiredness (20)<br>Unwell (11)<br>School/homework pressure (6)<br>Peer pressure (6)<br>Would rather do something else with spare time (27)<br>Concerns about appearance (17)<br>Concerns about exacerbating symptoms (25)<br>Concerns about the safety of physical activty with their condition (14)<br>Unclear what type/ intensity of physical activity to do (17)<br>Family concerns (17)<br>Other (10)

Appendix 4: Report Strengths and Limitations

⇒ Despite using widespread recruitment strategies, the number of respondents was small and centralised to South Wales, UK.

⇒ There is a high probability that those who did respond and attended the workshop are already advocates of the importance of discussions about physical activity, and exercise; this presents a high level of homogeneity within the data and limits the generalisability of the results.

⇒ The wide-ranging respondents from across the MDT is a strength, however the small number or respondents limits further generalisability.

Welsh Institute of Physical Activity, Health and Sport

WIPAHS is a pan-Wales network of all eight Welsh Universities and Sport Wales. With members based across Wales, we can capitalise on the nation's unique culture and its remarkable range of expertise, infrastructure, and facilities. WIPAHS brings together world-leading academics, with representatives from Sport Wales and Welsh Government, who are driven to answer practice-based questions, identify fundamental research questions, and ensure that findings are reflected in Welsh policy and practice. An advantage of working with WIPAHS is the access to such breadth of knowledge and resource available across the partners.

Our research expertise includes health inequalities and the use of physical activity as medicine. We are also experts in physical literacy, and the application of technology to promote physical activity or manage health conditions. Whilst working across the lifespan, many of our researchers are leading experts in using physical activity to improve short- and long-term outcomes in children and young people. Researchers have contributed to numerous Chief Medical Officer's physical activity and health expert working groups (including children and young people guidelines), and Physical Activity in the National Institute for Health Care Excellence (NICE) quality standards advisory committee for childhood obesity.

As a practice-driven organisation, WIPAHS seeks to answer the questions posed by partners working in the field, as well as widely disseminate knowledge across a diverse range of audiences. WIPAHS uses the transformative power of physical activity and sport to improve the lives of people in Wales.

Further information on projects we have led and supported can be found in our Annual Report 2023.

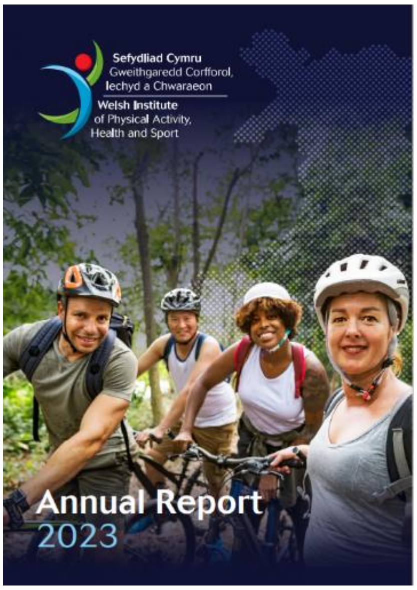

Sefydliad Cymru Gweithgaredd Corfforol, Iechyd a Chwaraeon

Welsh Institute of Physical Activity, Health and Sport

Contact Us

If you would be interested in discussing how the Welsh Institute of Physical Activity, Health and Sport (WIPAHS) can assist your organisation to answer important research and evaluation questions, or help provide your organisation with important insight, please do not hesitate to contact us to discuss.

Email: wipahs@swansea.ac.uk

Website: www.swansea.ac.uk/sports-science/astem/wipahs

@WIPAHSCymru

www.linkedin.com/in/wipahs
